# Improving Materials for a Technology-Based Childhood Obesity Intervention for Hispanic/Latino Families in Rural Nebraska: Mixed Methods Usability Test

**DOI:** 10.2196/84388

**Published:** 2026-10-29

**Authors:** Thais F Alves, Natalia Santos, Camila Fabiana Rossi Squarcini, Tzeyu L Michaud, Elisa Brosina De Leon, Fabio Almeida, Fabiana Brito

**Affiliations:** 1 Department of Health Services Research and Administration, College of Public Health, University of Nebraska Medical Center, 42nd and Emile, Omaha, NE, 68198-4350, United States, 1 402-559-2902; 2 Department of Health Promotion, College of Public Health, University of Nebraska Medical Center, Omaha, NE, United States; 3 Department of Health Sciences, Universidade Estadual de Santa Cruz, Ilhéus, Brazil; 4 Universidade Federal de Amazonas, Manaus, Brazil; 5 Department of Physical Education, Universidade Federal de Santa Catarina, Florianopolis, Brazil

**Keywords:** usability testing, user-centered design, childhood obesity, Hispanics, rural communities, Framework for Reporting Adaptations and Modifications-Enhanced, FRAME

## Abstract

**Background:**

Childhood obesity remains a public health concern in the United States, disproportionately affecting Hispanic/Latino and rural populations. Despite this disparity, there is a lack of culturally relevant programs specifically designed to reach this population. To address this gap, our team culturally adapted a technology-based childhood obesity intervention, Hispanic Family Connections (HFC), targeting Hispanic/Latino families living in rural areas. The intervention comprises 2 main components: interactive voice response (IVR) calls and a workbook with at-home activities to promote family engagement.

**Objective:**

The objectives were to (1) evaluate the usability of the IVR calls and workbook included in the HFC intervention and (2) document and plan adaptations to these components using the FRAME (Framework for Reporting Adaptations and Modifications-Enhanced).

**Methods:**

We conducted 5 mixed methods, task-based usability tests (UTs) using the think-aloud procedure. All 5 participants were women, self-identified as Hispanic/Latino, and were Spanish literate. Each participant completed 3 think-aloud tasks designed to evaluate the usability of the IVR calls and workbook chapters. Usability was assessed using the After-Scenario Questionnaire (ASQ) at the end of each task and the System Usability Scale (SUS) at the end of the UT session. All sessions were recorded, and data were organized into two primary categories: (1) quantitative results (ASQ and SUS) and (2) qualitative data (participants’ comments and researchers’ observations). Findings were compiled and analyzed following the 8 main domains of FRAME.

**Results:**

For the workbook, ASQ scores showed a high level of ease in task completion (mean 1.1, SD 0.32), satisfaction with completion time (mean 1.0, SD 0.00), and satisfaction with support information (mean 1.5, SD 0.97). For the IVR calls, participants reported a higher level of ease in task completion (mean 1.0, SD 0.00), satisfaction with completion time (mean 1.0, SD 0.00), and satisfaction with support information (mean 1.2, SD 0.45). SUS results were positive for 80% (4/5) of participants, with an average usability score above the benchmark (mean 75.5, SD 15.04). Using FRAME, we identified and documented 30 suggestions for adaptations and improvements to the workbook and IVR calls. The majority of suggestions were content-related (n=26, 86.67%), with most focusing on tailoring/tweaking, or refining existing materials.

**Conclusions:**

The UT results, combined with systematic documentation using FRAME, allowed us to identify and prioritize the adaptations needed to improve the intervention materials. These refinements will prepare the HFC intervention for implementation in a subsequent feasibility study.

## Introduction

Childhood obesity is a significant public health concern, disproportionately affecting Hispanic/Latino populations [1], with greater disparities in rural areas [2]. Reaching underserved Hispanic/Latino communities presents unique challenges due to geographic isolation, cultural differences, and limited access to resources, such as trained bilingual staff, designated spaces, and transportation [3,4]. While efforts have been made to develop programs addressing childhood obesity, as seen by the 172 studies identified in a 2024 review [5], there remains a shortage of culturally relevant and adapted programs that effectively engage Hispanic/Latino families in rural settings [6,7].

Leveraging technology can offer a promising strategy to overcome geographical and linguistic barriers and deliver targeted interventions [8] by bringing accurate and culturally appropriate information in the desired language to participants’ phones. The use of technology in rural health is not new and has vastly improved over time [9], as seen with the advancements in telecommunications [10] to more modern medical AI interventions [11]. However, to ensure the effectiveness of such interventions, it is crucial to evaluate their usability and make necessary adaptations to optimize their impact.

Usability can be defined as “the extent to which a product can be used by specified users to achieve specified goals with effectiveness, efficiency, and satisfaction in a specified context of use” [12,13]. Usability tests (UTs) are valuable tools that allow designers and researchers to understand how easy it is to use a product/resource in a specific context, and they should focus on the user’s experience [14]. Results are used to map opportunities for improvement and solving problems related to the design/product/resource being tested [15,16]. UTs can be a crucial method to achieve acceptability and the successful implementation of a technology [17,18]; therefore, their use could be vital in evaluating materials developed for public health interventions. By conducting usability testing during the program design phase, researchers can identify potential challenges and areas for improvement, thereby enhancing the program’s long-term sustainability. This is an especially crucial step for technology-based interventions as they require extensive planning and initial funding [19]. One model that can support researchers in the development of UTs is the FRAME (Framework for Reporting Adaptations and Modifications-Enhanced) [20]. The FRAME provides a comprehensive approach for documenting and reporting adaptations made to interventions [21]. This framework is applicable for usability testing, as it offers a structured way to track and plan the necessary changes to improve the intervention’s effectiveness.

Our research team developed the Hispanic Family Connections (HFC) [22] intervention to address the unique needs of Latino families residing in rural areas, such as the need for culturally and linguistically appropriate health programming. Recognizing the challenges faced by this population in accessing health care and partaking in healthy behavioral practices that can improve their children’s health, we designed HFC tailored to their specific cultural and local circumstances. The primary objective of this study was to evaluate the usability of interactive voice response (IVR) calls and the workbook, as part of the HFC intervention. As a secondary aim, we documented and planned the adaptations to the IVR calls and the workbook using the FRAME.

## Methods

### Study Design

This study used a mixed methods, task-based approach to evaluate the overall usability of IVR calls and a workbook. The materials evaluated are components of an intervention (ie, HFC) aiming to culturally adapt a technology-delivered, family-based childhood obesity prevention program for rural Hispanic/Latino families. The HFC focuses on parents as agents of change and includes 2 parent classes, followed by 10 IVR automated telephone support calls over 6 months (Table 1). Program materials are provided in a parent workbook with activities to be completed at home that align with IVR calls to promote increased physical activity and consumption of fruits and vegetables, in concert with decreased sugary-drink consumption and screen time [22]. The HFC is an adaptation of the Family Connections program, originally designed for English speakers [23].

**Table 1.** Examples of Hispanic Family Connections (HFC) interactive voice response (IVR) call content in English and Spanish.

IVR call type	English version	Content tested in Spanish
Call for goal setting	To set a goal to be consistent in helping your child do some physical activity every day this week, press 1. To set a goal to be consistent in offering a number of fruit and vegetable options at every dinner this week, press 2. To set a goal to plan for contingencies for after-school snacks that your child can choose from, press 3. To set a goal to be consistent in limiting the amount of television your child watches this week to less than 2 hours a day, press 4. If you would like me to repeat the goals, press star.	Para establecer el objetivo de ser coherente en ayudar a sus hijos a realizar actividad física todos los días esta semana, presione 1. Para establecer el objetivo de ser coherente en ofrecer una serie de opciones de frutas y verduras en cada cena de esta semana, presione 2. Para establecer el objetivo de un plan de contingencias para refrigerios después de la escuela que sus hijos puedan elegir, presione 3. Para establecer el objetivo de limitar el tiempo que sus hijos ven televisión esta semana a menos de 2 horas al día, presione 4. Si quiere que repita los objetivos, presione Asterisco.
Call for motivation	Way to go! It is so great that you were able to use your parenting skills to help your family make some changes. Keep up the great work! If you would like to hear some tips about how to keep on track with your parenting skills, press 1 now. If not, press 2 to move on to next week’s topic.	¡Muy bien! Es genial que haya podido usar sus habilidades para la crianza de los hijos para ayudar a su familia a realizar algunos cambios. ¡Siga con el buen trabajo que viene haciendo! Si desea escuchar algunos consejos sobre cómo continuar avanzando con sus habilidades de crianza, presione 1 ahora. De lo contrario, presione 2 para pasar al tema de la próxima semana.

All UTs were conducted using materials in Spanish, and we have translated some examples of the resources used exclusively to present in this article.

### Usability Testing of Intervention Materials

All intervention materials evaluated are available in Spanish as part of the cultural adaptation characteristic of this program. The Workbook is divided into 9 chapters, guiding parents through understanding the health impact of childhood obesity and providing education and activities to support behavioral and environmental changes. Examples of activities include learning how to read a nutrition fact label, planning physical activities as a family, role modeling positive behaviors, and reflecting on current shopping and eating habits (Figure 1). The Workbook also includes extra resources and supplemental materials, such as BMI tables and a guide on how to use the calls.

**Figure 1.**
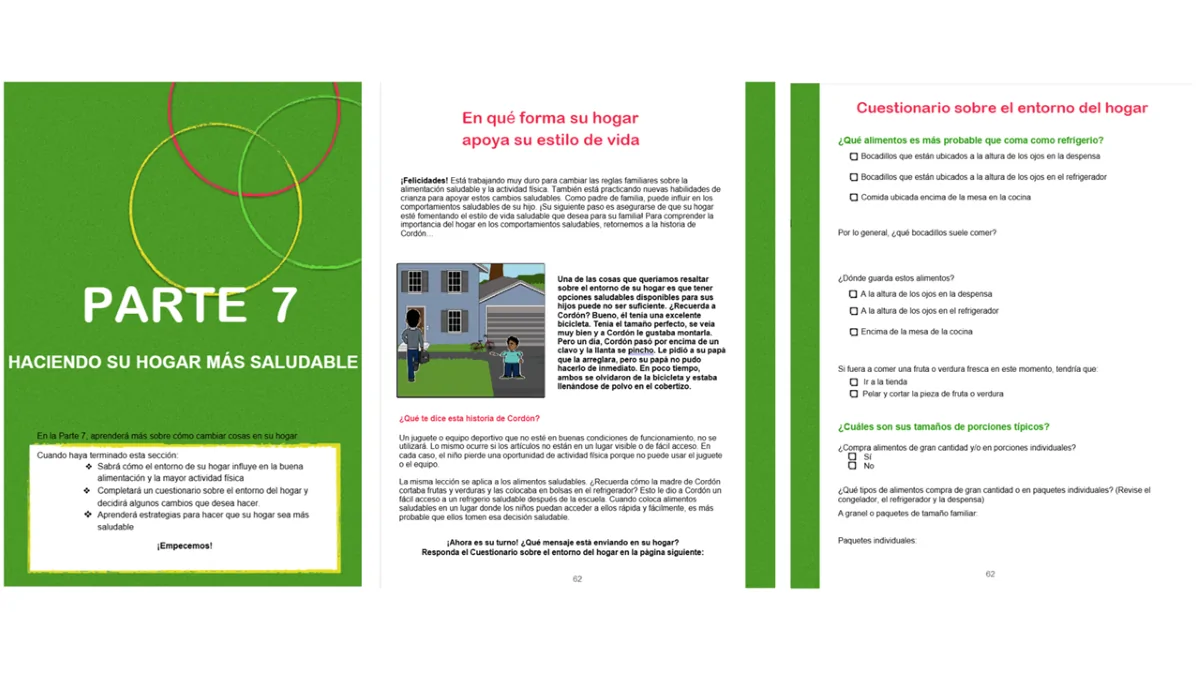
Example of the content and exercises from the Hispanic Family Connections (HFC) workbook, in Spanish. The interactive voice response (IVR)–automated telephone support calls follow the workbook’s content logic, and parents set and track goals for their family’s behavioral change using this technological support. During the 6-month period, while receiving the calls, parents can choose what to focus on, share their thoughts on their progress, and set new goals.

### Participants and Setting

Our recruitment goal was 5 to 10 participants, as recommended for this type of study [24]. Eligibility criteria for participation in this research included the following: parents/guardians/caregivers of children aged 6 to 14 years who were of Hispanic/Latino origin and literate in Spanish. The HFC research team partnered with 24 rural counties in Nebraska [22] to test the intervention’s feasibility throughout the study’s duration, and for the usability testing portion of the study specifically described in this paper, we had community health workers (CHWs) from 3 areas in rural Nebraska leading the recruitment efforts. Based on their client list and the study’s eligibility criteria, the CHWs contacted potential participants by phone and explained the research objectives and informed consent to those who were interested in participating. We conducted the UTs in a public library and in a meeting room at the health department. The places were selected and reserved by our CHW partners based on our requirements for a quiet location where we could set up the recording materials in their communities. More detailed information about the HFC design and materials can be found in the protocol paper [22].

### Process

We conducted UTs with 5 participants. Data collection occurred in May 2022, involving participants from 2 health departments in rural Nebraska. We applied the think-aloud method as a guide for conducting the UT because it allows testing observers to understand and track participants’ thought processes while navigating 1 randomly selected intervention session. To start the UT (process described in Figure 2), the trained, bilingual facilitator explained the activity and asked for the participant’s narrative consent. After agreeing to participate, they completed a short questionnaire exploring demographics and health literacy screening information [25]. We explained the think-aloud procedure in their preferred language (English or Spanish) and asked the participants to narrate their actions while they were performing tasks and to share their perceptions about the materials [14]. The facilitator led each session using a script developed by the research team with the support of an expert in user experience, while another staff team member acted as a notetaker. In some cases, a CHW was present to assist with the session facilitation.

**Figure 2.**
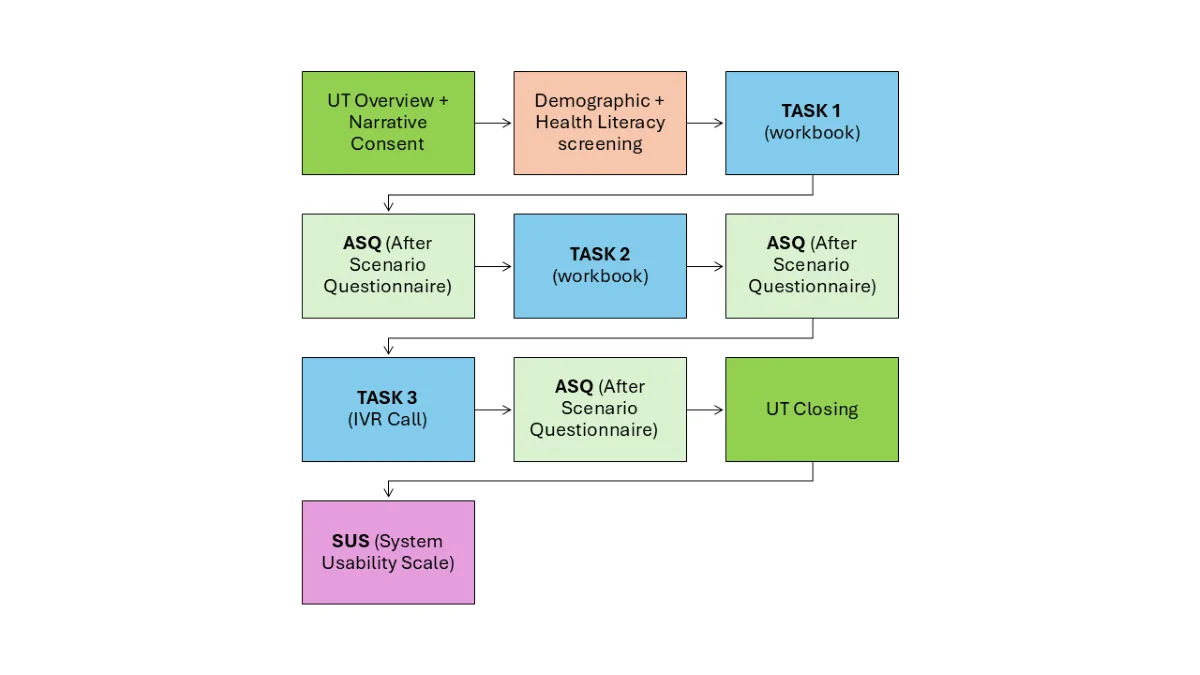
Usability test (UT) data-collection process per participant. The arrows indicate the sequence of steps taken by the research team and the tasks each participant executed. IVR: interactive voice response.

Each UT consisted of the following: we asked participants to perform 3 tasks, the first 2 related to the Workbook and the third involving an IVR call. Tasks included engaging with the IVR system, accessing the workbook, calculating BMI, and setting exercise goals for the family. The facilitator provided the workbook in Spanish and a smartphone (iOS operating system) for the participants to navigate and test the calls. Additionally, after each attempted task, we used the standardized posttask questionnaire called After-Scenario Questionnaire (ASQ) to measure user satisfaction [26,27]. At the end of the UT, participants answered the System Usability Scale (SUS) [27,28] to assess overall usability of the materials (Figure 2). Each session was video recorded on Zoom and transcribed using NVivo (Lumivero) software.

### Quantitative Measures

Composed of 3 items, the ASQ [26] was used after every task to evaluate participants’ satisfaction regarding the ease of completing the task, the amount of time it took, and the support information to complete it. It contains a Likert scale from 1 (strongly agree) to 7 (strongly disagree). In this way, a lower score indicated a higher level of satisfaction with usability [26]. By the end of the test, participants responded to the 10-item SUS [28], that evaluates usability considering aspects related to effectiveness, efficiency, and satisfaction. The Likert scale goes from 1 (strongly disagree) to 5 (strongly agree). The score for odd questions (Q1, Q3, Q5, Q7, and Q9) is the scale position minus 1. For even questions (Q2, Q4, Q6, Q8, and Q10), the score is 5 minus the scale point. The overall value of SUS was calculated by multiplying 2.5 by the sum of the scores [27,28], and the classification ranges were as follows: worst (0‐25), poor (25.01‐52.00), ok (52.01‐72.74), good (72.75‐85.57), and excellent (85.58 or more) [29,30]. We chose these 2 specific scales because they are objective, easy to understand, and reliable [27]. Both questionnaires are widely used by researchers when evaluating user experience [31].

### Qualitative Adaptations Report

We used the FRAME [21] as a base for the data integration in this mixed methods research. We compiled the findings emerged from the UT within the 8 aspects of FRAME: (1) when the modification occurs; (2) if the modifications were planned (proactive or reactive); (3) who participated in the decision to modify; (4) what is modified; (5) at what level of delivery the modification is made; (6) nature of the content modification; (7) fidelity-consistency of the modification; and (8) reasons for the modification [21]. The FRAME framework has its roots in implementation science, which is why we chose this tool to help translate the research findings into practice, tracking, and planning the necessary adaptations to the HFC materials evaluated.

### Data Analysis

To address the 2 objectives proposed in this mixed methods study, we organized the data in two categories for integration and analysis: (1) data from the standardized questionnaires (ASQ and SUS) and (2) data from participants’ comments (think-aloud procedure) and researchers’ notes (the facilitator and notetaker) during the UT. For the descriptive analysis, average and SD were used for numerical variables, and relative and absolute numbers for categorical variables were used for the demographic questionnaires and scores on the SUS and ASQ.

For more in-depth analysis of the think-aloud data, we transcribed the UT-recorded sessions using NVivo software and uploaded the researchers’ notes to build a robust dataset. The materials were coded based on the FRAME [21] framework, with the primary aim of tracking and planning the necessary adaptations to the IVR calls and workbook. The researchers created a Microsoft Excel sheet containing the main aspects of the framework, adding the time necessary to complete each task and the total time spent on the UT. Conducting the UT using a mixed methods approach allows for tracking and planning improvements based not only on the questionnaires’ results but also on analyzing participants’ body language, visible comfort or discomfort, and time spent on specific tasks.

### Ethical Considerations

This study was approved by the University of Nebraska Medical Center Institutional Review Board (#0745‐20-EP) and is available for consultation [32] (identifier: NCT04731506). Narrative informed consent was obtained for the study, and each individual had the right to refuse to participate, answer any questions, or stop the UT at any time. We offered the participants a US $25 gift card for time compensation. To maintain participants’ anonymity during translation, data analysis, and results description, we referred to them in our materials as “staff,” followed by a study identification number.

## Results

The participants were all women (N=5, 100%), with a mean age of 41.25 (SD 6.08) years, and all were born in Latin America. Furthermore, the majority completed elementary school (n=3, 60%), were married (n=4, 80%), and were employed part-time (n=2, 40%). Regarding health literacy, most participants were highly confident in filling out Spanish medical forms by themselves (n=3, 60%) instead of the English version (0%; Table 2).

**Table 2.** Characteristics of the usability-test participants (N=5).

Variable	Value
Age (y), mean (SD)	41.25 (6.08)
Gender, n (%)	
Women	5 (100)
Country of birth, n (%)	
Mexico	2 (40)
Cuba	1 (20)
El Salvador	1 (20)
Guatemala	1 (20)
Education, n (%)	
Elementary	3 (60)
High school	1 (20)
College	1 (20)
Marital status, n (%)	
Married	4 (80)
Not married	1 (20)
Employment, n (%)	
Employed for wages, part-time	2 (40)
Employed for wages, full-time	1 (20)
Homemaker	1 (20)
No response	1 (20)
Health literacy, n (%)	
How confident are you filling out English medical forms by yourself?	
1 (extremely)	0 (0)
2 (quite a bit)	1 (20)
3 (somewhat)	1 (20)
4 (a little)	0 (0)
5 (not at all)	2 (40)
No response	1 (20)
How confident are you filling out Spanish medical forms by yourself?	
1 (extremely)	3 (60)
2 (quite a bit)	1 (20)
3 (somewhat)	1 (20)
4 (a little)	0 (0)
5 (not at all)	0 (0)
Since you speak another language besides English at home, we want to know in your opinion, how well you speak English. Would you say, you speak English:	
1 (well)	0 (0)
2 (very well)	0 (0)
3 (not well)	2 (40)
4 (not at all)	3 (60)

The SUS provides a subjective assessment of usability, with questions presented on a Likert scale, ranging from 1 (strongly disagree) to 5 (strongly agree). Overall, results were positive for 80% (4/5) of participants, with usability average above the score (75.50, SD 15.04; Table 3). When analyzing the SUS items individually, a clear trend was observed in the responses. Positive statements (odd-numbered items) predominantly received ratings between “agree” and “strongly agree.” Negative statements (even-numbered items) were mostly rated between “disagree” and “strongly disagree.” These results suggest a favorable perception among participants regarding the usability of the evaluated material.

**Table 3.** System Usability Scale (SUS) questionnaire results^a^.

SUS response by question^b^^c^	Participants					Mean (SD)
	1	2	3	4	5	
Q1. I think that I would like to use this system frequently	5	5	1	5	3	3.80 (1.79)
Q2. I found the system unnecessarily complex	1	1	1	1	3	1.40 (0.89)
Q3. I thought the system was easy to use	5	5	5	5	3	4.60 (0.89)
Q4. I think that I would need the support of a technical person to be able to use this system	5	5	N/A^d^	5	5	5.00 (0.00)
Q5. I found that the various functions in this system were well integrated	5	4	4	5	5	4.60 (0.55)
Q6. I thought there was too much inconsistency in this system	1	1	1	2	4	1.80 (1.30)
Q7. I would imagine that most people would learn to use this system very quickly	5	5	5	5	4	4.80 (0.45)
Q8. I found the system very cumbersome to use	1	1	1	1	3	1.40 (0.89)
Q9. I felt very confident using the system	5	4	5	5	5	4.80 (0.45)
Q10. I needed to learn a lot of things before I could get going with this system	1	3	1	4	5	2.80 (1.79)

a Total SUS score: mean 75.50 (SD 15.04).

b Odd questions: positively worded items.

c Even questions: negatively worded items.

d N/A: no response.

Participants were satisfied with the workbook (mean 1.2, SD 0.61) and IVR (mean 1.1, SD 0.26), as shown by the ASQ results in Table 4. For the workbook, participants were satisfied with the ease of task completion (mean 1.1, SD 0.32), completion time (mean 1.0, SD 0.00), and support information (mean 1.5, SD 0.97). Likewise, participants expressed high satisfaction with the IVR system, particularly regarding the ease of task completion (mean 1.0, SD 0.00), the time required to complete tasks (mean 1.0, SD 0.00), and the usefulness of the support information provided (mean 1.2, SD 0.45).

**Table 4.** After-Scenario questionnaire (ASQ) results by materials.

Variables	Mean score (SD)
Workbook	
Q1. Overall, I am satisfied with the ease of completing the tasks in this scenario.	1.1 (0.32)
Q2. Overall, I am satisfied with the amount of time it took to complete the tasks in this scenario.	1.0 (0)
Q3. Overall, I am satisfied with the support information (online help, messages, documentation) when completing the tasks.	1.5 (0.97)
Total	1.2 (0.61)
IVR**^a^**	
Q1. Overall, I am satisfied with the ease of completing the tasks in this scenario.	1.0 (0)
Q2. Overall, I am satisfied with the amount of time it took to complete the tasks in this scenario.	1.0 (0)
Q3. Overall, I am satisfied with the support information (online help, messages, documentation) when completing the tasks.	1.2 (0.45)
Total	1.1 (0.26)

a IVR: interactive voice response.

After tracking the necessary adaptations and using FRAME elements to organize the workbook and IVR call adaptations and modifications, we identified a total of 30 suggestions (Table 5). All suggestions were associated with the preimplementation phase (n=30, 100%), were mostly considered reactive (n=26, 86.67%), and were made by CHWs (n=13, 43.33%). Moreover, the suggestions were bound with the “content” (n=26, 86.67%), focusing especially on “tailoring/tweaking/refining” (n=13, 43.33%) to improve the “individual and group levels” (n=26, 86.67%) and other levels (n=4, 13.33%). Another point analyzed, extracted from FRAME, was the different goals of the modifications, such as “addressing cultural factors” (n=11, 36.67%), “improving fit with recipients” (n=5, 16.67%), and combining “improving fit with recipients” with “addressing cultural factors” (n=5, 16.67%).

**Table 5.** Adaptations tracked for the workbook and interactive voice response (IVR) calls using the FRAME (Framework for Reporting Adaptations and Modifications-Enhanced) framework.

Elements of FRAME	Value (%)
1. When did the modification occur?	
Preimplementation	30 (100)
Implementation	0 (0)
Scale up	0 (0)
Maintenance	0 (0)
2. Were the adaptations planned?	
Proactive	4 (13.33)
Reactive	26 (86.67)
3. Who suggested the modification?	
CHW^a^	13 (43.33)
Recipient	2 (6.67)
Researcher	8 (26.67)
Researcher notetaker	7 (23.33)
4. What is modified?	
Content	26 (86.67)
Contextual	4 (13.33)
Training and evaluation	0 (0)
Implementation and scale-up activities	0 (0)
5. What was the nature of modifications?	
Adding elements	7 (23.33)
Format	4 (13.33)
Substituting	6 (20.00)
Tailoring/tweaking/refining	13 (43.33)
6. What was the level of delivery?	
Clinic/unit level	1 (3.33)
Individual/target intervention group	26 (86.67)
Target intervention group	3 (10)
7. What was the goal?	
Improve effectiveness/outcomes	1 (3.33)
Improve fit with recipients	5 (16.67)
Improve fit with recipients/to address cultural factors	5 (16.67)
Increase reach or engagement/improve effectiveness/outcomes	2 (6.67)
Increase reach or engagement/increase retention	2 (6.67)
Increase retention/improve feasibility/improve fit with recipients	1 (3.33)
Increase satisfaction	1 (3.33)
To address cultural factors	11 (36.67)
To address cultural factors/increase satisfaction	2 (6.67)
8. Modifications suggested and adoption status	
Delivery, modified (yes or no)	
First visit in-person (n=1)	No
Participant would like to have in-person meetings every 3 calls or every 2 months, to understand the family context and evaluate the process (n=1)	No
Slow down voice calls (options), for better understanding (n=1)	Yes
Teach participants how to use their cell phone for calls, on the first in-person meeting (n=1)	Yes
All calls are in English, need to translate (n=1)	Yes
Create magnetic/erasable option for grocery planning (n=1)	Yes
Inclusion of content, modified (yes or no)	
Include calorie list of foods (most consumed foods or Latinos/Hispanics such as tortillas; n=1)	No
“Local resources” need to be adapted to what is available in the area (n=1)	No
Replace “web” by “redes sociales” (n=1)	Yes
To use “camicata” instead of “senderismo” (n=1)	Yes
To include a brief explanation about what self-esteem is (n=1)	No
Add photo examples of each food group (n=1)	Yes
Review entire page, participants struggling to understand and respond (n=1)	Yes
Include the option “don‘t know,” in “actividad de rutina” (n=1)	No
Questions “cuantos dias a la semana?” and “cuantos minutos?” need to be finalized (n=1)	Yes
Workbook - pg 65 - replace “hot dogs” with “Perro caliente” (n=1)	Yes
Use “comidas para llevar” instead of “comidas al paso” (n=1)	Yes
Replace “Cordon” with “Luis” (n=1)	Yes
Review all figures, some are still in English and need to be translated (n=1)	Yes
To create a space at the end of the workbook to add the contact of the CHW and the research team (n=1)	Yes
Layout, modified (yes or no)	
Review page layout (n=3)	Yes
Review figure (n=2)	Yes
Review the content, there is a word missing; and the sentence below the figure is out of context (n=1)	Yes
Less color on food planning (n=1)	Yes
Center text “overweight” (n=1)	Yes
Change name to “Conexiones Familiares” (n=1)	No
Staff previous training, modified (yes or no)	
Add training with CHW on how to navigate the workbook (n=1)	Yes
Overall modifications, n (%)	
No	6 (20)
Yes	24 (80)

a CHW: community health worker.

Analyzing the suggested modifications, 23.33% (7/33) were not adjusted because there were 3 changes to the fidelity of the materials and the intervention (eg, participants would like to have in-person meetings every 3 calls or every 2 months to understand the family context and evaluate the process; change the name to “Conexiones Familiares” for the workbook and calls; include a brief explanation about what is self-esteem in page 4 of the workbook), 1 was already part of the intervention (first visit-in-person) and 2 individualized the materials and feasibility trial (eg, insert in the workbook the “local resources” need to be adapted to what is available in the area; include in chapter 5 of the workbook a calorie list of foods, most-consumed foods among Latinos/Hispanics, such as tortillas).

## Discussion

### Principal Findings

This UT mixed methods study’s primary objective was to evaluate the usability of IVR calls and a workbook of HFC, and to document and plan adaptations to these materials. The ASQ results revealed the ease of completing the task, satisfaction with the time required to complete the task, and satisfaction with the support information. The ASQ, a widely used instrument by researchers, has been published in studies to analyze the usability of applications [33-35] and a telerehabilitation system [36], a patient-centered website [37], and others. However, no studies have used ASQ to assess the usability of printed workbook materials and IVR focusing on overweight children by the time of this publication. The SUS showed good usability of the workbook and the IVR calls, primarily when it refers to participants’ ability to quickly learn how to use the system (Q7), respondents’ confidence in using the system (Q9), and whether the system is simple (Q2) and friendly (Q8). The SUS, like the ASQ, is widely used to evaluate the usability of a product. The SUS has been applied to analyze a social robot-based platform to prevent childhood obesity [38], a mobile app offering techno–health care services and assistance with adhering to a Mediterranean diet [39], and a risk estimation tool for obesity prevention [40]. However, the studies are not targeted at Hispanic/Latino children who live in rural areas of the United States.

Advancing to the FRAME coding results, it is clear what the nature and stage of the research are, considering that all adaptations are in the preimplementation phase, and the majority are related to content improvement—wording, figures, layout. When looking at the adaptation’s nature and goals, it is explicit that the challenge and importance of working with Hispanics/Latinos from different backgrounds are significant. Our participants included Latinos from 4 different countries living in rural Nebraska, and even among Spanish heritage, there are multiple cultural differences within communication and language preferences that impose a need for a more neutral accent and grammar in the intervention materials.

There are multiple dimensions to consider when working on cultural adaptation for Hispanics or Latinos, especially for public health interventions. Therefore, interventions that include cultural adaptation processes, such as the HFC, must go beyond translation. The materials need to incorporate cultural expressions, using language and images that recipients can relate to; they must be meaningful, consider the context and reach, and reflect the target population [41,42]. Therefore, cultural adaptation should be ongoing and regularly refined to address evolving needs and preferences within Hispanic/Latino communities [43].

Another interesting fact we noticed during data analysis and result description was the number of adaptations that emerged from participants (recipients) when compared to the CHWs and the researchers. Only 6.67% (2/30) of the suggestions were made by the participants, and they were all contextual, such as having in-person meetings every 2 months, thereby showing more concern about the intervention itself than about the material specifications. Based on this, we highlight the importance of choosing the think-aloud procedure [14] for this study, and having the UTs video recorded, and aggregating the researcher notes to NVivo as part of the dataset and coded based on FRAME [21].

Sometimes, in technological studies, the participants may not think something needs to be changed or improved because it is their first contact with that type of resource. However, for the research team, it is important to draw on all relevant background and experience with the topic and develop the skills to conduct an in-depth analysis of qualitative and quantitative outcomes. Also, it is important to consider that some populations, specifically minorities, may think their opinions are not important or they do not share them freely for other reasons and barriers imposed by different contexts [44-46]. Therefore, when working with studies such as cultural adaptations, inclusive language, or anything related to minorities and health disparities, it can be essential to conduct mixed methods approaches independently of the sample, since data integration can provide specific, in-depth, essential information for data analysis [47-49].

Of the 30 adaptations tracked by our team using FRAME, 7 were not addressed immediately in this phase of the study. One of them was not addressed because it was already part of the program, having the first meeting in person, and the others because they could affect fidelity, that is, consistency with the intervention’s core elements. It is important to note that these UTs are part of the HFC. Therefore, any future adaptations that involve changes in fidelity will be assessed after the analysis of the HFC feasibility study’s results and before planning the intervention scale-up for a broader audience.

### Strengths

The methodological innovation proposed by the research team in this study is noteworthy. In technology studies (especially usability and satisfaction assessments), it is new to aggregate standardized questionnaires (such as the ASQ and SUS) with frameworks from other disciplines, such as the FRAME framework that we chose for the adaptations report and coding. Our goal in the incorporation of FRAME into the UT analysis is to help facilitate translation into practice, since this framework’s roots are in the precepts of Implementation Science, which is essential when working with technological applications for public health interventions, thereby permitting us to have a broader vision of the process of adaptation and improving delivery and effectiveness.

### Limitations

This study assessed the usability of the workbook and IVR, but we did not test the effectiveness of the intervention materials at this point. The results of this study indicate that participants evaluated our research materials positively. Task-based tests were conducted in controlled research settings, a different context from using the materials in real life. This requires the research team to be open to ongoing feedback from participants.

Also, the participants are from 2 different counties in rural Nebraska; all are women, and only Spanish-literate individuals were eligible, conditions that do not represent the diversity of Hispanics/Latinos we intend to reach with the intervention in the future. Our exclusively female sample may also limit the generalizability of our findings. This may be attributed to women being more directly connected with our recruiters or having flexible schedules to participate in the research activities. Childhood obesity interventions such as these are family-based; therefore, understanding the roles and perspectives of fathers is crucial for long-term success.

### Conclusions

By testing the usability of the workbook and IVR calls, this study addressed part of the gap related to the development of culturally relevant interventions for Hispanic/Latino families living in rural areas. Our results from the SUS and ASQ show that the usability of HFC materials, before adaptations, was rated mostly as good. For both resources evaluated (the workbook and IVR calls), participants demonstrated higher satisfaction with the level of ease of task completion. Of the 30 adaptations tracked using FRAME, we addressed 23, primarily related to content changes, such as wording, layout, and figure adjustments. By understanding the strengths and limitations of the intervention materials, we aimed to enhance their accessibility and cultural relevance, and possibly their future effectiveness when applied in a feasibility study. The UT results, combined with the systematic documentation using FRAME, allowed us to identify and prioritize the adaptations needed to improve the intervention materials. These refinements will prepare the HFC intervention for implementation in a subsequent feasibility study.

## Abbreviations

ASQ: After-Scenario Questionnaire
CHW: community health worker
FRAME: Framework for Reporting Adaptations and Modifications-Enhanced
HFC: Hispanic Family Connections
IVR: interactive voice response
SUS: System Usability Scale
UT: usability test
